# Analysis of the relapse of imported *Plasmodium vivax* and *Plasmodium ovale* in five provinces of China

**DOI:** 10.1186/s12936-023-04642-y

**Published:** 2023-07-13

**Authors:** Hui Yan, Shujiao Wei, Yuan Sui, Shenning Lu, Weiwei Zhang, Xiangyang Feng, Ying Liu, Tao Zhang, Wei Ruan, Jing Xia, Wen Lin, Benedikt Ley, Sarah Auburn, Shizhu Li, Jun Li, Duoquan Wang

**Affiliations:** 1 Guangxi Zhuang Autonomous Region Center for Disease Control and Prevention, Nanning, 530028 China; 2 Brown School, Washington University, St. Louis, MO USA; 3 National Institute of Parasitic Diseases, Chinese Center for Disease Control and Prevention (Chinese Center for Tropical Diseases Research); NHC Key Laboratory of Parasite and Vector Biology (National Institute of Parasitic Diseases, Chinese Center for Disease Control and Prevention), 200025 Shanghai, China; 4 Henan Provincial Center for Disease Control and Prevention, Zhengzhou, 450016 China; 5 Anhui Provincial Center for Disease Control and Prevention, Hefei, 230601 China; 6 Zhejiang Provincial Center for Disease Control and Prevention, Hangzhou, 310051 China; 7 Hubei Provincial Center for Disease Control and Prevention, Wuhan, 430079 China; 8 Global and Tropical Health Division, Menzies School of Health Research and Charles Darwin University, Darwin, NT Australia; 9 Chinese Center for Disease Control and Prevention, National Institute of Parasitic Diseases, Chinese Center for Tropical Diseases Research, WHO Collaborating Centre for Tropical Diseases, National Center for International Research on Tropical Diseases, Ministry of Science and Technology, Key Laboratory of Parasite and Vector Biology, Ministry of Health, School of Global Health, Chinese Center for Tropical Diseases Research, Shanghai Jiao Tong University School of Medicine, Shanghai, 200025 China

**Keywords:** Imported, *Plasmodium vivax*, *Plasmodium ovale*, Relapse or recrudescence, Incubation, Primaquine, Interval

## Abstract

**Background:**

The global battle against malaria is facing formidable challenges, particularly in controlling *Plasmodium vivax* and *Plasmodium ovale*, whose cases have not been reduced as effectively as *Plasmodium falciparum* because of their relapse. This study investigates the current situation and underlying factors contributing to relapse or recrudescence of imported cases of *P. vivax* and *P. ovale*, and seeks to provide a reference for reducing relapse or recrudescence in malaria-free areas and offers a scientific basis for designing strategies to prevent imported re-transmission.

**Methods:**

This study analysed imported *P. vivax* and *P. ovale* in Anhui, Zhejiang, Henan, Hubei, and Guangxi provinces during 2014–2021 by retrospective analysis. A case–control study was conducted on patients who experienced relapse or recrudescence.

**Results:**

From 2014 to 2021, 306 cases of *P.vivax* and 896 cases of *P.ovale* were included in the study, while 75 cases had relapse or recrudescence, including 49 cases of *P. ovale* (65.33%) and 26 cases of *P. vivax* (34.67%). Within less than 5 weeks after returning to the country, 122 cases of *P. vivax* (39.87%, 122/306) and 265 cases of *P. ovale* (29.58%, 265/896) occurred. Within less than 53 weeks, the ratio of *P. vivax* was 94.77% (290/306), and that of *P. ovale* was 89.96% (806/896). Among the cases experiencing relapse or recrudescence, only 1 case of *P. vivax* (1/26 3.85%) and 3 cases of *P. ovale* (3/49 6.12%) occurred within less than 5 weeks after the first onset, whereas 21 cases of *P. vivax* (21/26 80.77%) and 42 cases of *P. ovale* (42/49 85.71%) occurred within less than 53 weeks after the first onset. The difference in relapse or recrudescence due to different drugs and medication regimens and medical activities at various levels of medical institutions was statistically significant.

**Conclusion:**

In areas where malaria has been eliminated, routine health screening in a scientific time frame for people returning from at-risk areas can effectively improve the efficiency of preventing re-transmission, thereby reducing prevention costs and disease burden. Preventing patients from self-treating and strengthening medication regulations in health facilities are key measures to reduce relapse or recrudescence.

## Background

Global malaria cases experienced a significant increase of 14 million in 2020 compared to 2019, with a corresponding increase of 69,000 deaths [1]. The global fight against malaria is facing severe challenges. Notably, global malaria control efforts have significantly reduced *Plasmodium falciparum* cases, but to a lesser extent in *Plasmodium vivax* and *Plasmodium ovale* [2, 3]. Consequently, the proportion of *P. vivax* and *P. ovale* among total malaria cases is increasing. Available evidence suggests that stable transmission of *P. vivax* occurs in many areas south of the Sahara [4]. The infection of *P. ovale* is endemic to tropical West Africa [5] and is found in several parts of sub-Saharan Africa [6].

The failure to significantly reduce the infection or incidence of *P. vivax* and *P. ovale* can be partly attributed to the relapse properties of *P. vivax* and *P. ovale* [7, 8]. Intrahepatic hypnozoites cause malaria relapse and patients reappear with symptoms weeks or even years after cure, while residual Plasmodium causes recrudescence in the peripheral blood [9].China’s National Malaria Elimination Surveillance Programme has established a surveillance programme to prevent the re-transmission of imported malaria. There is an explicit requirement to screen for *Plasmodium* in people travelling with patients who have developed the disease after returning from endemic areas abroad [10]. However, there are no clear guidelines on the timing and frequency of *Plasmodium* screening for clinically cured patients and their close contacts. Despite the collaborative efforts of 13 ministries in China, including the entry–exit inspection and quarantine association malaria prevention and control measures have not been included in the quarantine declaration. Although the last domestic case of *P. vivax* Yunnan Province was reported in 2016, hundreds of imported *P. vivax* cases continue to be reported every year [11]. Similarly, the incidence of imported *P. ovale* cases is increasing each year [12]. The presence of the *Anopheles* vector further amplifies the risk of re-establishing imported malaria, relapse, or recrudescence cases [13].

The presence of *P. ovale* is widespread and is considered a relapsing form of malaria, although evidence for liver hypnozoites is insufficient. However, evidence from *P. vivax* strongly suggests the existence of liver hypnozoites [14]. Similar to other countries [15, 16], relapse cases of imported *P. vivax* have also been reported in China [17]. Using molecular biology techniques, Imwong et al. demonstrated that recurrent *Plasmodium* and first-episode *Plasmodium* genotypes were both homologous and non-homologous, making it challenging to strictly distinguish between reinfection, relapse, and recrudescence [18, 19]. Endemic areas lack specific prevention and control measures for relapse or recrudescence, as well as lack a systematic assessment of the clinical presentation, treatment, and recrudescence characteristics of *P. ovale* cases [20]. However, in non-endemic areas, it is possible to exclude epidemic background interference and more accurately observe the relapse or recrudescence of *P. ovale* and *P. vivax*. This study conducted a descriptive analysis of imported *P. vivax* and *P. ovale* in five provinces of Guangxi, Zhejiang, Anhui, Henan, and Hubei from 2014 to 2021. It was performed a case–control study of the relapse or recrudescence cases to analyse their basic conditions, intervals of relapse or recrudescence, and related influencing factors. Findings from this study provide a reference for reducing relapse or recrudescence in malaria-free areas and a scientific basis for formulating effective measures to prevent imported re-transmission.

## Methods

### Data collection and source

The data were collected from the China Infectious Disease Reporting System (CIDRY) and Parasitic Diseases Information Reporting Management System (PDIRMS). For the study, 417 imported cases of *P. vivax* and 1066 imported cases of *P. ovale* were collected from Zhejiang, Anhui, Henan, Hubei and Guangxi from 1 to 2014 to 31 December 2021. Of these, 306 cases of *P. vivax* and 896 cases of *P. ovale* were included in the study after excluding patients with mixed infections, unknown dates of the return, or unknown information of first onset. The episode of each period after returning to China was analy*s*ed. Furthermore, the interval of relapse or recrudescence and the potential external influencing factors were analy*s*ed.

### Data information

Malaria data from five provinces were reviewed, which included *Plasmodium* species, basic demographic information (age, gender), place of infection, date of return, date of onset, date of first and last dose, drugs used, and treatment institutions. In this study, relapse or recrudescence was not strictly distinguished and was combined for analysis.

### Definition of imported malaria, the first episode, recrudescence, relapse, self-treatment

Imported malaria was defined as: (i) a history of travel to malaria-endemic areas abroad 1 month before onset of symptoms, or malaria cases with clear overseas infection and no evidence of local transmission. It should be noted that the determination of whether the patient contracted malaria abroad relied on whether the patient has received malaria treatment abroad or has corresponding clinical symptoms and self-medication. The absence of evidence of local transmission was determined by combining the absence of local primary or imported secondary cases in the patient’s current residence in the past 5 years, the presence of suitable *Anopheles* mosquito vectors and the results of active monitoring of residents around the patient’s home. It should be noted that the determination of whether the patient contracted malaria abroad relied on the patient’s own recall. The absence of evidence of local transmission was determined by combining the malaria incidence in the patient’s current residence over the past 5 years, monitoring of *Anopheles* mosquito vectors at the epidemic point, and active monitoring of residents around the patient’s home. (ii) The county-level Centers for Disease Control and Prevention (CDC) of the place where the case is reported shall be completed within epidemiological investigation of the case within 3 days, collecting evidence of foreign infection and excluding evidence of local infection [10]. Additionally, a country was considered as a place of infection for imported cases if an individual had a travel history to an endemic malaria area and developed symptoms within 1 month of returning [21].

The following specification were used for the first episode, relapse or recrudescence and self-medication: (i) Due to some patients’ inability to recall the onset and duration of treatment as well as the name of the medication used abroad, the first episode after returning to China was defined as the initial attack. (ii) The patient in the study did not leave China after returning home until the occurrence of the second episode, and epidemiological investigation could rule out the possibility of a new infection. (iii) When the same case had multiple episodes, the study analysed whether the type of drugs used in the first treatment influenced the occurrence of the second episode and assessed the time interval between the two episodes. Only the second episode was included in the study. (iv) Self-medication was defined as patients bringing medication from abroad upon their return to their home country and using it based on personal experience at the time of onset.

### Diagnosis of malaria

Malaria was diagnosed based on a combination of epidemiological history, clinical manifestations, and laboratory tests. Microscopy was used by county and city CDCs patient to detect the presence of malaria parasites. Patients identified through clinic visits or malaria-risk population screening underwent a detailed epidemiological investigation by the CDC to determine the nature of the infection, such as imported, local infection, or re-emergence. Medical institutions used microscopy or rapid diagnostic tests (RDTs) for diagnosing malaria cases, and the reported cases were subsequently reviewed by the CDC at the same level. Confirmed or suspected cases reported by counties and cities were finally confirmed by provincial malaria diagnostic reference laboratories using microscopy and polymerase chain reaction (PCR) [22].

### Treatment for patients

Once diagnosed with malaria, patients with mild cases were given a course of oral medication in an outpatient setting, while those with severe cases were hospitalized. The treatment of *P. vivax* and *P. ovale* followed the National guideline of the technical regulations for application of antimalarials (WS/T485-2016) in China. For adults, the recommended medication and doses were: (i) Chloroquine phosphate orally at 600 mg (4 tablets) on the first day, followed by 300 mg (2 tablets) on the second and third day, together with primaquine phosphate (7.5 mg/tablet, 24 tablets in total; 3 tablets/day for 8 days). (ii) The dose of piperaquine phosphate plus primaquine phosphate and the duration of treatment were the same as chloroquine phosphate plus primaquine phosphate. (iii) Oral dose of dihydroartemisinin–piperaquine (dihydroartemisinin 40 mg, piperaquine 171.4 mg/tablet) was administered for 8 tablets in total, with 2 tablets for the first dose and 2 tablets for each of 8 h, 24 h, and 32 h, together with primaquine phosphate (7.5 mg/tablet, 24 tablets in total; 3 tablets/day for 8 days). (iv) oral artesunate–amodiaquine (artesunate 100 mg, amodiaquine 270 mg/tablet) was administered for 6 tablets in total, with 2 tablets/day for 3 days, concomitantly with primaquine phosphate (7.5 mg/tablet, 24 tablets in total, 3 tablets each for 8 days). (V) Oral artemisinin piperaquine (artemisinin 62.5 mg, piperaquine 375 mg/tablet) was administered at a total of 4 tablets, with 2 tablets/day for 2 days. The cases of *P. vivax* and *P. ovale* required anti-relapse therapy with primaquine phosphate (7.5 mg/tablet, 24 tablets in total; 3 tablets each for 8 days) in the following spring (Table 1) [23]. According to the national guideline (WS/T485-2016) [23] in China, oral chloroquine was recommended for 3 days and primaquine was prescribed for 8 days for the treatment of P. vivax or *P. ovale* peripheral blood asexuals. If oral medication was not available, a 7 days continuous course of artesunate for injection was used as an alternative drug treatment. Therefore, less than 10 days interval between two episodes was considered as a recent treatment failure and categorized as the same episode.

**Table 1:** The treatment of *P. vivax* and *P. ovale* in China

Drugs	Specification and dosage	Medication method
Chloroquine phosphate	1200 mg, 8 tablets; 150 mg/tablet	4 tablets on the first day, 2 tablets on the second and third day; orally
Piperaquine phosphate	1200 mg, 8 tablets; 150 mg/tablet	4 tablets on the first day, 2 tablets on the second and third day; orally
Dihydroartemisinin–piperaquine	8 tablets; each tablet contained 40 mg dihydroartemisinin and 171.4 mg piperaquine phosphate	2 tablets for the first dose, 2 tablets for each of 8 h, 24 h, and 32 h; orally
Artesunate amodiaquine	6 tablets; each tablet contained 100 mg artesunate and 270 mg amodiaquine	2 tablets/day, 3 days; orally
Artemisinin piperaquine	4 tablets; each tablet contained 62.5 mg artemisinin and 375 mg piperaquine	2 tablets/day, 2 days, orally
Primaquine phosphate	180 mg, 24 tablets; 7.5 mg/tablet	3 tablets/day, 8 days, orally

### Case–control study

In this study, a case–control study was conducted as part of the overall descriptive study. Among the descriptive results, 75 cases of relapsed or recrudescence malaria were identified. These 75 cases were assigned as the experimental group, and each case was matched to 4 controls. The selection of case and control groups were ensured by epidemiological investigation and laboratory diagnosis. The case group was defined as patients with at least two malaria episodes after their return, while the control group had only one episode. Controls were matched accordingly to reduce potential confounding about age, sex, malaria species, and source of infection. As some controls could not meet the 1:4 standard, the final sample size consisted of 75 cases and 289 controls.

The inclusion criteria for the case–control study were as follows: (i) all recurrent or relapse cases of *P. vivax* and *P. ovale* reported in the five provinces from 2014 to 2021; (ii) controls were matched to cases by age, gender, *Plasmodium*, and source of infection.

The exclusion criteria for the case–control study were as follows: (i) those with incomplete information regarding the date of return and first onset of infection; (ii) those with mixed infections with other *Plasmodium*; (iii) those with mixed infections with *P. vivax* and *P. ovale*.

### Statistical analysis

Data collection and organization were performed using Microsoft Excel 2016 (Microsoft Corporation, USA) software. Descriptive analyses were conducted to examine the general information and epidemiological characteristics of all included cases. Statistical analyses were performed using the Statistical Package for Social Sciences (SPSS) 17.0 (IBM, USA) and GraphPad Prism 9 software (GraphPad Software, USA) from International Business Machines (IBM). The incubation period from the return to the first episode and the interval from treatment to the second episode were expressed as median (*M*) and interquartile range (IQR). The type of medication and the rate of relapse or recrudescence after treatment at different levels of medical institutions in case–control studies were tested by the chi-square test. The Fisher exact probability test was used when the number of grid frequencies was less than 5. Logistic regression analysis was conducted to analyse the effect of treatment and drug type on relapse or recrudescence at different levels of medical institutions. A *P*-value < 0.05 was considered statistically significant.

## Results

### Episode of imported cases of *P. vivax* and *P. ovale* after returning home

From 2014 to 2021, 306 cases of imported *P. vivax* and 896 cases of imported *P. ovale* were analysed from five provinces in China. Within less than 5 weeks after returning home, 122 cases of *P. vivax* (39.87%, 122/306) and 265 cases of *P. ovale* (29.58%, 265/896) occurred. Within less than 25 weeks, the *P. vivax* ratio was 77.12% (236/306), and the *P. ovale* ratio was 68.42% (613/896). Within 1 year of return (< 53w), the *P. vivax* ratio was 94.77% (290/306), and the *P. ovale* ratio was 89.96% (806/896). The longest incubation period observed was 175 weeks for *P. vivax* and 265.71 weeks for *P. ovale*. The median (*M*) and interquartile range (*IQR*) of the incubation period of cases after returning to the country until the first onset of symptoms was 9.14 weeks (*IQR* 3.14–24.57) for *P. vivax* and 12.43 weeks (*IQR* 3.79–30.50) for *P. ovale* (Table 2).

**Table 2:** The incubation period for the first episode of imported *P. vivax* and *P. ovale* and the interval of relapse or recrudescence from 2014 to 2021 (weeks)

	First episode		Relapse or recrudescence	
	*P. vivax*	*P. ovale*	*P. vivax*	*P. ovale*
Time of onset, n (%)				
< 5	122 (39.87%)	265 (29.58%)	1 (3.85%)	3 (6.12%)
5–12	61 (19.93%)	188 (20.98%)	5 (19.23%)	5 (10.2%)
13–24	53 (17.32%)	160 (17.86%)	5 (19.23%)	16 (32.65%)
25–52	54 (17.65%)	193 (21.54%)	10 (38.46%)	18 (36.73%)
53–78	11 (3.59%)	46 (5.13%)	5 (19.23%)	3(6.12%)
79–103	4 (1.31%)	9 (1%)	0	0
> 104	1 (0.33%)	35 (3.91%)	0	4(8.16%)
M	9.14	12.43	30.43	25.43
IQR	3.14–24.57	3.79–30.50	13.72–48.00	15.86–44.00
Mix	0.14	0	4.29	4.43
Max	175	265.71	69	234.43

Furthermore, 75 cases of relapses or recrudescence were observed during the same period. Within less than 5 weeks of return, there was 1 case (1/26, 3.85%) of *P. vivax and 3 cases* (3/49, 6.12%) of *P.oval****e***. Within less than 25 weeks, the *P. vivax* ratio was 42.31% (11/26), and the *P. ovale* ratio was 48.98% (24/49). Moreover, within 1 year of return (< 53w), the *P. vivax* ratio was 80.77% (21/26), and the *P. ovale* ratio was 85.71% (42/49). The longest interval time was 69 weeks for *P. vivax* and 234.43 weeks for *P. ovale*. The median (*M*) and interquartile range (*IQR*) from the treatment of the first onset to relapses or recrudescence was 30.43 weeks (IQR 13.72–48.00) for *P. vivax* and 25.43 weeks (IQR 15.86–44.00) for *P. ovale* (Table 2).

### Epidemiological characteristics of case and control groups in case–control studies

From 2014 to 2021, among 306 cases of *P. vivax* and 896 cases of *P. ovale*, we observed 75 relapses or recrudescence. Of these, 26 cases were of *P. vivax* (34.67%, 26/75) and 49 cases were of *P. ovale* (65.33%, 49/75). These cases were distributed among the provinces as follows: 5 cases in Anhui Province, 4 cases in Hubei Province, 16 cases in Henan Province cases, 18 cases in Zhejiang Province, and 32 cases in Guangxi Zhuang Autonomous Region. Moreover, of these 75 cases, 72 were males (96.0% 72/75) and 3 were females (4.0% 3/75). The minimum age of these cases was 27 years, the maximum age was 58 years, and the median age (M) was 51 years. On the other hand, of the 289 cases in the control group, which included 93 cases of *P. vivax* (32.2%, 93/289), 196 cases of *P. ovale* (67.82%, 196/289), the corresponding number of cases in the five provinces was 20, 16, 63, 54 and 136. Of these 289 cases, 280 were males (96.89%, 280/289), and 9 were females (3.11%, 9/289) (Table 3).

**Table 3:** Epidemiological characteristics of cases in case–control studies

Number of patients (100%)	Relapse or recrudescence		Control group	
	*P. vivax*	*P. ovale*	*P. vivax*	*P. ovale*
	n = 26 (%)	n = 49 (%)	n = 93 (%)	n = 196 (%)
Province				
Anhui	2 (7.69)	3 (6.1)	8 (8.6)	12 (6.1)
Zhejiang	6 (23.1)	12 (24.5)	22 (23.7)	32 (16.3)
Hubei	2 (7.69)	2 (4.1)	8 (8.6)	8 (4.1)
Henan	7 (26.9)	9 (18.4)	28 (30.1)	35 (17.9)
Guangxi	9 (34.6)	23 (46.9)	27 (29.0)	109 (55.6)
Ages				
21–30	4 (15.4)	4 (8.2)	23 (24.7)	24 (12.2)
31–40	8 (30.8)	15 (30.6)	26 (28.0)	66 (33.7)
41–50	10 (38.5)	14 (28.6)	33 (35.5)	70 (35.7)
51–60	4 (15.4)	16 (32.7)	11 (11.8)	36 (18.4)
Gender				
Male	25 (96.2)	47 (96.0)	90 (96.8)	190 (97.0)
Female	1 (3.8)	2 (4.0)	3 (3.2)	6 (3.0)
Infection area				
Asia	9 (34.6)		32 (34.4)	
Oceania	1 (3.8)		3 (3.2)	
East Africa	9 (34.6)	3 (6.1)	36 (38.7)	12 (6.1)
West Africa	3 (11.5)	19 (38.8)	8 (8.6)	76 (38.8)
Central Africa	4 (15.4)	24 (49.0)	14 (15.1)	96 (49.0)
Southern Africa		3 (6.1)		12 (6.1)

**Fig. 1:**
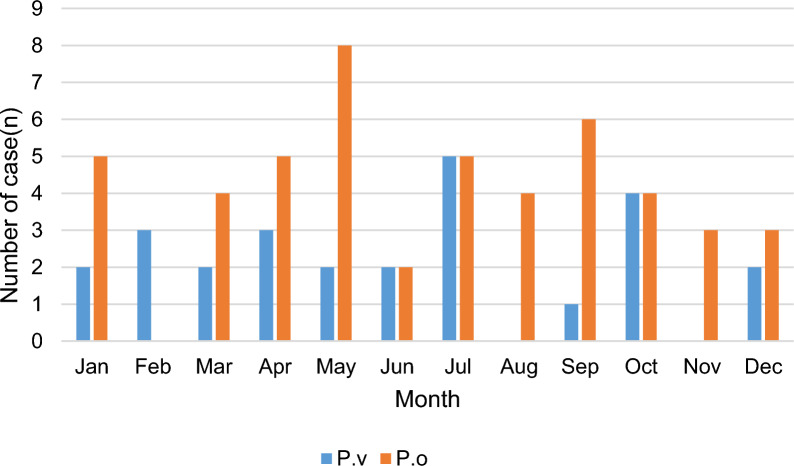
Monthly distribution of relapse or recrudescence cases of imported *P. vivax* and *P. ovale* from 2014 to 2021

The number of recrudescence or relapse cases in the case group from January to December was 7, 3, 6, 8, 10, 4, 10, 4, 7, 8, 3, and 5, respectively, with no significant seasonality (Fig. 1).

**Fig. 2:**
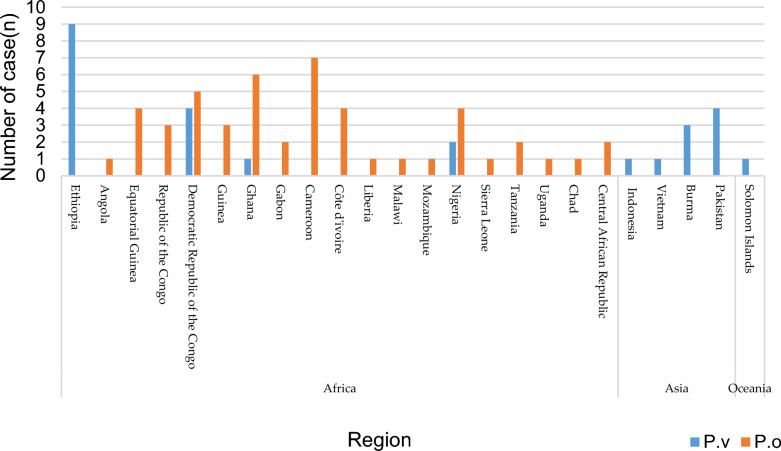
Sources of relapse or recrudescence cases of imported *P. vivax* and *P. ovale* from 2014 to 2021

The import of the case group originated from 24 countries, 19 of which were African (79.17%, 19/24), 4 Asian (16.67%, 4/24), and 1 Oceanian (4.17%, 1/24). Most of the imported cases of *P. vivax* came from Ethiopia, while all *P. ovale* cases were from Africa (Fig. 2).

### Effect of drug type on relapse or recrudescence

After the clinical cure of the first episode, 26 cases of relapse or recrudescence of *P. vivax* (8.50%, 26/306) and 49 cases of relapse or recrudescence of *P. ovale* (5.47%, 49/896) were observed. Although the difference in relapse or recrudescence rate between them was not statistically significant (χ^2^ = 3.575, *P* > 0.05), the difference in relapse or recrudescence rate between different drug groups was statistically significant (χ^2^ = 35.737, *P* < 0.001). Specifically, a statistically significant difference was observed between the “chloroquine and artemisinin and primaquine” drug group and all non-primaquine drug groups (*P* < 0.05). On the other hand, no statistically significant difference was found between the “artemisinin and primaquine” group and all other drug groups (Table 4).

**Table 4:** Comparison of factors influencing relapse or recrudescence of imported *P. vivax* and *P. ovale* from 2014 to 2021

Influencing factors	Relapse or recrudescence	Control group	Accounted for (%)	χ^2^	*P*-value
Drugs				35.737	< 0.001
a Artesunate for injection	15	29	34.09		
a ACTs	29	83	25.89		
a ACTs and artemisinins for injection	6	13	31.58		
a, b Artemisinins and primaquine	3	20	13.04		
b Chloroquine and artemisinins and primaquine	7	138	4.83		
Different levels of medical institutions				75.319	< 0.001
c Self-treatment	23	5	82.14		
d Outpatient clinic	10	69	12.66		
d County-level medical institutions	16	123	11.51		
d City-level medical institutions	17	65	20.73		
d Provincial medical institutions	9	27	25.00		
Any history of treatment abroad				1.038	0.308
Received treatment abroad	63	251	20.06		
No treatment abroad	12	33	26.67		

At the 0.05 level, there was no significant difference between group a, no significant difference between group b, and a significant difference between group a and b; no significant difference between group c, no significant difference between group d, and a significant difference between group c and d

Among the drug type factors, there were 15 cases without accurate medication records in the relapse or recrudescence group and 5 cases in the control group. Among the extraterritorial infection history factors, there were 5 cases with missing information in the control group

### Treatment outcomes of medical institutions at all levels

The study revealed significant differences in the overall relapse or recrudescence rate of patients after treatment with various methods, including self-administered medication, outpatient treatment, county-level medical institutions, city-level medical institutions, and provincial-level medical institutions (χ^2^ = 75.319, *P* < 0.001). Moreover, the differences between self-administered medication and all levels of medical institutions were statistically significant (*P* < 0.05); however, the difference between different medical institutions was not statistically significant (Table 4).

### History of overseas treatment

There was no significant difference in the rate of relapse or recrudescence, with or without a history of overseas treatment before returning (Table 4).

### Multi-factor analysis of relapse or recrudescence

**Fig. 3:**
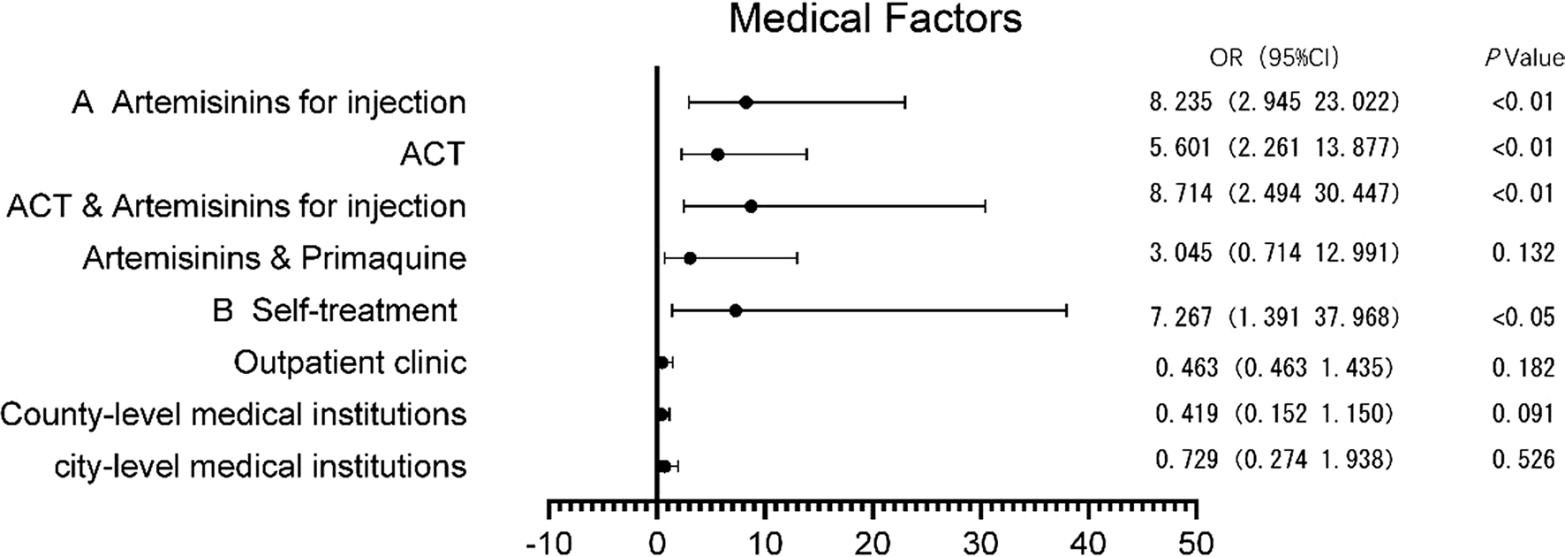
Multifactor analysis of the relapse or recrudescence of imported *P. vivax* and *P. ovale* from 2014 to 2021. A indicates drug groups, and B indicates medical institution groups

A multifactorial analysis of the effect of the treatment drug and the treatment institution on relapse or recrudescence was performed, using “chloroquine and artemisinin and primaquine” and “provincial medical institution” as references for the drug and treatment institution groups, respectively. In addition to the “artemisinin and primaquine” group, the “artemisinin-based combination oral and injectable artemisinin” (β = 2.165), “artesunate for injection” (β = 2.108), “artemisinin-based combination oral” (β = 1.723) were statistically significant (*P* < 0.01) for relapse or recrudescence. On the other hand, the “self-administered” group exhibited statistical significance (*P* < 0.05) (β = 1.983) for treatment facilities, while no statistical significance was found among medical institutions (Fig. 3).

## Discussion

Imported malaria poses a significant challenge to malaria control efforts in low-endemic or eliminated countries and regions [24]. Instances of local transmission caused by imported cases or unidentified infections have been reported [25–27]. Therefore, effective monitoring of imported cases of malaria is essential to prevent local infection. This study shows that a substantial proportion of individuals infected with *P. vivax* and *P. ovale* develop malaria within the first month (< 5w) of returning to non-endemic areas, with nearly 40% of *P. vivax* and 30% of *P. ovale* patients, resulting in a peak incidence period. Over the course of the first 6 months, the ratio among these individuals increases to approximately 70%, indicating a second peak period. These results highlight the importance of implementing robust malaria surveillance measures targeting individuals who may be at risk due to similar work or travel histories abroad and who return alongside diagnosed patients. Additionally, health reminders should be provided to these individuals to raise awareness of the importance of malaria surveillance. Therefore, it was proposed that at-risk individuals should be screened for *Plasmodium* once a week for the first month after their return, once a month from the second to the 6th month, and once every 6 months from  7th to 24th month. This strategy would allow for the timely detection of potential sources of infection, improve the timeliness of prevention, and reduce control costs and disease burden.

Various factors contribute to the relapse or recrudescence of *P. vivax*, including patient compliance with treatment, defective treatment regimens, and resistance of *Plasmodium* to anti-malarial drugs [28]. This study revealed that the relapse or re-emergence rates of *P. vivax* and *P. ovale* in five provinces are 8.50% and 5.47%, respectively. These findings are consistent with earlier research on *P. vivax* [29], and even 77% of secondary cases in some places were due to relapse of *P.vivax* [30]. The occurrence of multiple relapses play a significant role in sustaining malaria transmission in endemic regions [31]. The time interval between the initial onset of *P. vivax* infection and relapse is related to the number of sporozoites having infected the patient [32–37]. However, no technology is currently available to detect the presence of hypnozoites in the liver of clinically cured patients and predict relapse timing. The median time from treatment of the first onset to relapses or recrudescence was 30.43 weeks (*IQR* 13.72–48.00) for *P. vivax* and 25.43 weeks (*IQR* 15.86–44.00) for *P. ovale*. These findings highlight the importance of conducting health follow-ups after clinical cure to monitor for relapse or recrudescence. Based on the results, follow-up screening of malaria patients at 6 months to 1 year after treatment to reduce the costs of prevention and control efforts caused by premature follow-up screening. This approach can effectively detect relapse or recrudescence and reduce re-transmission risk.

The findings also uncovered that the type of drug and the patient’s self-administration behaviour was associated with relapse or recrudescence of *P. vivax* and *P. ovale*. Daher et al. [38] showed that the difference in efficacy between the combination of chloroquine-primaquine and artemisinin-based combination + primaquine was not statistically significant. These evidence further confirm the importance of primaquine’s ability to effectively eliminate hypnozoite in the liver cells in *P. vivax* and *P. ovale* infections [39]. However, the occurrence of relapse after primaquine treatment is still unknown [40]. Notably, individuals with CYP2D6 (isotype of cytochrome P450 [CYP450]) polymorphisms have been shown to have slower primaquine metabolism [41]. CYP2D6 is naturally polymorphic, with a widely present variant carried by approximately 25% of the world’s population. This variant is associated with a significant decline in the primaquine efficacy, putting individuals carrying this variant at a higher risk of relapse [42, 43]. No statistical differences were found between “artemisinin and primaquine” and other drug groups. This may be attributed to factors such as primaquine dosage used, or whether or not the patient carried the CYP2D6 variant.

According to China’s National guideline of the technical regulations for application of anti-malarials (WS/T485-2016), chloroquine plus primaquine is the recommended anti-malarial drug for *P. vivax* and *P. ovale*. In cases where chloroquine is ineffective, alternative drugs, such as piperaquine, rorannadine phosphate, or artemisinin-based combinations in association with primaquine can be used [23]. However, the study revealed inconsistencies in drug use across all five provinces, primarily due to the challenges in procuring chloroquine phosphate and primaquine nationwide from 2019 to 2021. Inadequate knowledge of treatment standards among attending physicians in medical institutions also contributed to inconsistent drug usage. Zhang et al. [44] highlighted delayed diagnosis in 30.08% of imported malaria cases in five provinces of China from 2014 to 2021, indicating clinicians’ limited sensitive towards malaria management. Similarly, a study on malaria-related deaths in the USA from 1963 to 2001 demonstrated that more than half of the deaths were due to faulty healthcare, primarily resulting from clinician errors [45]. The high rate of relapse or recrudescence (82.14%) observed after self-treatment of the initial malaria episode further emphasizes the importance of providing health education to individuals at-risk after returning from malaria-endemic areas, discouraging self-treatment practices. In this study, county and municipal-level medical institutions were the primary providers of treatment for malaria patients. As outbound labourers primarily resided within the rural areas and towns under county-level jurisdiction, accessibility to county-level medical institutions was higher. Therefore, strengthening and consolidating malaria treatment capacity at the county level is crucial for maintaining a malaria-free status.

Despite the significant findings, there are certain limitations to acknowledge in the study. Firstly, some key information relied on patients’ self-narratives, such as medications taken and the exact duration of treatment, which could be subject to inaccuracies. Secondly, there was a lack of technical means to accurately distinguish between relapse or recrudescence. Additionally, this study did not distinguish between the two genetically distinct subspecies of *P. ovale curtisi* and *P. ovale wallikeri*.

## Conclusions

In areas where malaria has been eliminated, implementing health follow-up visits for people who may be at risk of infection due to travel from endemic areas can be an effective strategy for preventing re-transmission and reducing the associated costs and disease burden. Preventing patients from self-treating and strengthening medication regulation in health facilities are critical measures to reduce relapse or recrudescence.

## Data Availability

The dataset analysed during the current study is available from the corresponding authors upon reasonable request.
